# Sleep problems and injury risk among juveniles: A systematic review and meta-analysis of observational studies

**DOI:** 10.1038/s41598-017-10230-3

**Published:** 2017-08-29

**Authors:** Yun-Bing Wang, Zhen-Lang Guo, Fan Zhang, Yong Zhang, Shu-Sheng Wang, Yong Zhao

**Affiliations:** 1 Graduate School, Chongqing Medical University, Chongqing, 400016 China; 2 Department of Hepatobiliary Surgery, The Second Affiliated Hospital of Chongqing Medical University, Chongqing, 400010 China; 3 The Second Clinical College, Guangzhou University of Chinese Medicine, Guangzhou, 510405 China; 4 School of Public Health and Management, Chongqing Medical University, Chongqing, 400016 China; 5 Department of Urology, Guangdong Provincial Hospital of Chinese Medicine, Guangzhou, 510120 China

## Abstract

Recent studies have reported inconsistent results regarding the association between sleep problems and injury risk among juveniles. Moreover, the extent of this risk remains largely unexplored. Thus, a systematic review and meta-analysis was conducted by our team to determine whether sleep problems increase the incidence of injuries among juveniles. PubMed, PsycINFO, Embase, and Cochrane Library databases were searched for relevant studies that explored the association between sleep problems and injury risk and have been published before July 2016. Multivariate adjusted odds ratio (OR) and associated 95% confidence intervals (CIs) were extracted and pooled using random-effects models. A total of 10 observational studies involving 73,418 participants were identified. Meta-analysis findings suggested that juveniles with sleep problems held a 1.64 times higher risk of injury than that of juveniles without sleep problems (OR: 1.64, 95% CI: 1.44–1.85). This relationship was also supported by subgroup analyses, which were based on different countries and study designs. The current evidence indicates that sleep problems are significantly associated with injury risk among juveniles. Sleep problems are highly important for young people; hence, sleep researchers and occupational physicians should focus on this aspect. Nevertheless, high-quality and adequately powered observational studies are still needed.

## Introduction

In juveniles, injuries are frequent and can be related to sleep difficulty, which has gradually become a major problem worldwide^1^. In particular, sleep problems among juveniles may cause serious problems in their physical and emotional health, academic success, and safety^2, 3^. Globally, around 830,000 juveniles (nearly 2300 persons daily) die annually from injuries^4^. To reduce the number of injured juveniles, scholars must determine the associated risk factors. This knowledge suggests an important role for preventive measures.

Sleep problem, a juvenile health concern, is a potentially relevant risk factor for injuries. Several recent studies have shown that sleep problems are associated with injury risk among juveniles^5–7^. Indeed, sleep interruption results in various adverse consequences, including obesity, increased mortality, and diabetes^8, 9^. Juveniles are physically more likely to suffer than other age groups from chronic sleep deprivation because of the steady-state regulation of maturation changes during the circadian and sleep-wake cycle, leading to chronic sleep debt^10, 11^.

Suspicions on the hazard in injury risk originally arose from the routine investigations of juveniles with sleep problems in the USA^12^. Subsequently, cross-sectional studies of juveniles in the China and France also found increased risks of injury^13, 14^, but have not been confirmed in other investigations^15, 16^. However, to our knowledge, no review or meta-analysis has explored the influence of sleep problem on injuries among juveniles globally. Hence, our study aimed to examine such relation. A broad systematic review and meta-analysis of published studies was performed to precisely evaluate the relationship between sleep problems and injuries among juveniles, and help health-care professionals make related clinical decisions. This study complied with the recommendations of the Cochrane Collaboration, and our study protocol was registered as *PROSPERO 2016: CRD 42016051257*.

## Methods

### Search strategies

This study followed the recommendations of the Cochrane Collaboration^17^. For reporting, we adhered to the guidelines developed by the Meta-analysis of Observational Studies in Epidemiology (MOOSE) group^18^.

To identify eligible studies, we performed a comprehensive literature search in the electronic databases of PubMed, PsycINFO, Embase, and Cochrane Library from database inception up to July 2016. Each database was searched using a combination of Medical Subject Headings (MeSH) and non-MeSH terms without restrictions to the geographical region, publication status, and language. Manual search techniques were also employed to identify appropriate studies (manual searches of reference lists were performed). The main search was completed independently by the investigators. Any discrepancy was resolved by consulting an investigator not involved in the initial procedure.

### Study selection

#### Study types

Observational studies (prospective and retrospective cohort studies, cross-sectional studies, and case-control studies) that explored the relationship between sleep problems and injuries among juveniles were included without limitations to geographical region, publication status, or language. Selected studies were these published in English and containing original data (excluding reviews), as well as those with frequency data for sleep disturbances of any type or severity, diagnosed sleep disorders, or specific sleep problems/symptoms. Studies were excluded if any of the following factors were identified: (1) studies without detailed results and (2) animal studies.

#### Definitions of sleep problems, injury, and participants

The nature and quantity of sleep in juveniles differ from those of adults. In this review, the risk factors we considered were sleep problems of any frequency, duration, and severity. Accordingly, all sleep disorders described in the International Classification of Sleep Disorders (ICSD-2) were considered, including sleep quantity, sleep medication, and daytime sleepiness^19^. In this review, studies that primarily focused on all injury types in juveniles were included. Specifically, studies that explored the relationship between sleep problems and work-related injuries were excluded. Therefore, studies that only reported commercial motor vehicle or ship and aircraft crashes were not included. The participants of the included studies were juveniles of both genders with ages not exceeding 18 years old.

### Data extraction

Two reviewers independently extracted the data by using a predefined data extraction form. Disagreements were resolved by discussion or consensus with a third reviewer. The following data were extracted: first author, study characteristics (i.e., year, duration, setting, and design), participant characteristics (i.e., mean age, sample size, and exposure), and risk estimates (odds ratios [ORs] or relative risks [RRs]) with corresponding 95% confidence intervals (CIs). For studies with insufficient information, the primary authors were contacted if possible to acquire and verify the data.

### Methodological quality assessment

Assessment was separately conducted by two independent reviewers (YBW and ZLG) in accordance with the modified version of the Newcastle–Ottawa Scale (NOS) for quality assessment of observational studies^20^. All disparities between the two reviewers were resolved with consultation with another expert in the field. The following domains were used to evaluate the methodological quality of the observational studies: selection and comparability of study groups and exposure/outcome of interest. Each numbered item within the categories of selection and exposure/outcome was awarded with a maximum of one star. Across studies, a maximum of two stars can be given for comparability. High-quality papers reached 60% or more of the maximum number of stars^21^.

### Statistical analyses

Pooled analysis was conducted using Stata 12.0 to assess the relationship between sleep problems and injuries among juveniles. The reported risk estimates in each study were re-analyzed to work with consistent definitions. The I-square (I^2^) test was performed to assess the effect of study heterogeneity on the meta-analysis results. According to the Cochrane review guidelines, a severe heterogeneity of I^2^ ≥ 50% warrants the use of random-effects models. Otherwise, a fixed-effects model would be used. Egger’s regression coefficient determination and visual inspection of the funnel plot were also performed to assess publication bias if a sufficient number of studies (n ≥ 10) were included in this research. Statistical significance was set at P < 0.05^22^. A subgroup analysis on the basis of different study designs and countries were conducted to explore the possible origins of heterogeneity. Subgroup analyses were also performed to obtain additional information regarding the risks people might hold under different conditions.

## Results

### Study characteristics and methodological quality

**Figure 1:**
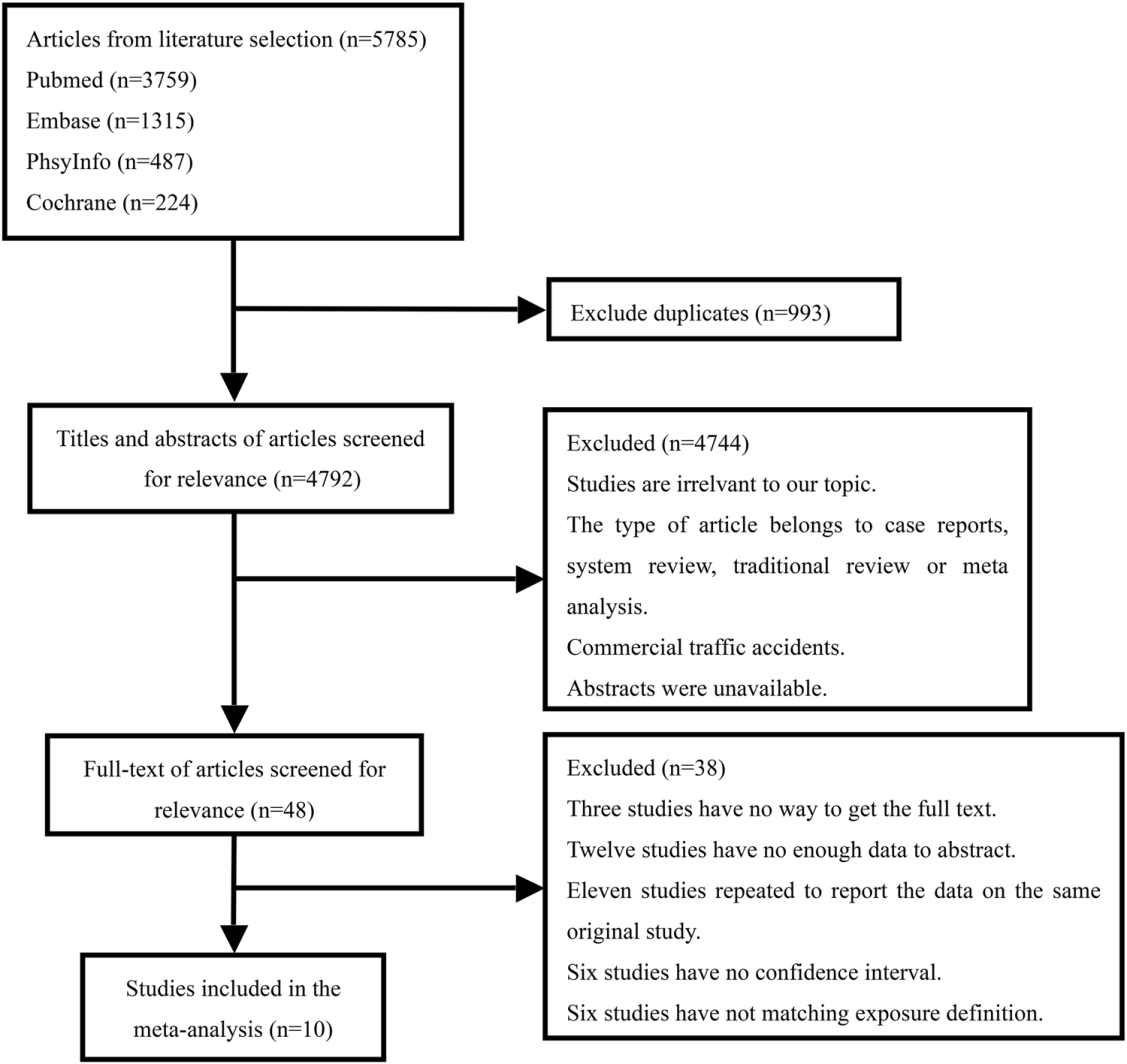
Flow diagram of study selection. Note: A total of 5785 studies was identified through our search of the four electronic databases. After removing 993 duplicates, only 4792 studies were retrieved. After reading the titles and abstracts, only 48 studies remained. A total of 38 articles were excluded after reading the full-text.

A flow chart depicting the search process and study selection is shown in Fig. 1. A total of 5785 studies was identified through our search of the four electronic databases. After removing 993 duplicates, only 4792 studies were retrieved. After reading the titles and abstracts, only 48 studies remained. A total of 38 articles were excluded for the following reasons. Three studies did not provide a full text, 12 studies contained insufficient data for extraction, 11 studies repetitively reported data from the same original study, 6 studies did not present CIs, and 6 studies did not match the exposure definition.

A total of 10 observational studies^12–16, 23–27^ were included in the meta-analysis. The basic characteristics of the included studies are described in Table 1. These studies (8 cross-sectional studies and 2 case-control studies) were published between 2006 and 2015 and involved 73,418 participants. In the included clinical studies, the sample sizes varied between 112 and 61,696 participants.

**Table 1:** Basic characteristics of the included studies.

Author, year	Study design (quality)	Sample (Location)	Population (Mean age or age range in years)	Definition of injury	Definition of sleep problem
Milewski *et al.* ^12^	Cross-sectional study (high)	54 male, 58 female (USA)	School athletes (15.2 ± 1.5; 12–18 y)	Sports related injury during the previous and upcoming year (Q)	< 8 hours of sleep per night
Chau *et al.* ^13^	Cross-sectional study (high)	778 male, 781 female (France)	Urban area student population (13.5 ± 1.3)	Single and repeated school and out-of-school injuries during the present school year (Q)	Having trouble going to sleep or staying asleep/being dissatisfied with sleep
Tan *et al.* ^14^	Cross-sectional study (high)	2,347 male, 2,633 female (China)	School-aged children (12.7; 9–18 y)	Injury was restricted to unintentional injury, which was caused by something or someone without purpose and consciousness, defined as an accident that restricted normal activities for at least four hours or required medical attention, caused loss of consciousness, loss of awareness, or loss of memory for any length of time (Q)	< 8 hours of sleep on average during the past month (including napping time)
Jaung *et al.* ^15^	Cross-sectional study (low)	1551 (China)	Rural area student population (7th –9th)	Road traffic injuries were defined as injuries incurred as a result of a road traffic collision involving at least one vehicle in motion on a public or private road that results in at least one person being injured (Q)	Difficulty going to sleep on school nights/Awakened at night and had trouble going back to sleep/Had nightmares
Lam *et al.* ^23^	Cross-sectional study (high)	1429 (China)	Adolescent population attending the first 3 years of high school (13–17 y)	Unintentional injuries were defined as any injuries that were not deliberately caused by the student or another person the past 3 months (Q)	< 7 hours of sleep per night
Boto *et al.* ^24^	Case-control study (high)	1101 (Portugal)	Children population (1–14 y)	Accidental falls led to contusion/hematoma, open wound, fracture (Q)	< 8 hours of sleep in the preceding week
Kim *et al.* ^16^	Cross-sectional study (low)	30,810 male, 30,886 female (Korea)	Korean adolescents population (12 –18 y)	Including accidents from riding a bicycle to commute to school, slips and falls in the classroom, corridors, the ground, toilets, stairs, and other places and tooth fractures due to exercise or accident over the previous 12 months (Q)	< 7.5 hours of sleep per night
Stallones *et al.* ^25^	Cross-sectional study (high)	146 male, 116 female (USA)	Adolescents who lived on farms in Colorado (13–18 y)	Information was obtained on any injury that occurred in the 12 months before the interview date that required ≥ 4 hours of restricted activity or medical attention, and the total number of injuries occurring within the year regardless of severity. (Q)	Adolescents ( < 9.25 or < 8.5) were asked to describe usual sleep habits (what time they go to bed and what time they go to sleep on school days and on weekends, and what time they wake up on school days and on weekends).
Pizza *et al.* ^26^	Cross-sectional study (high)	339 (Italy)	Adolescents who had a driver’s license and attended 1 of 7 high schools in Bologna, Italy (18.4 ± 0.6; 18–21 y)	Moto vehicle crashes (Q)	Nocturnal sleep habits and symptoms suggesting sleep disorders (un-refreshing sleep, sleep disordered breathing, restless legs syndrome, hypnagogic hallucinations, sleep paralysis, sleepwalking, sleep terror, and insomnia), and a subjective report of daytime sleepiness.
Li *et al.* ^27^	Case-control study (high)	389 (China)	Rural school-aged children (6–13 y)	Injuries incurred by the children were then grouped into external cause categories as defined in Chinese epidemiologic studies, including falls, cut/pierce, overexertion, struck, burns, foreign body, transportation accidents, unintentional poisoning, animal-related injuries, drowning/submersion, electrical injuries, and others according to the International Classification of Disease(Q).	Specific sleep pattern and sleep problems were assessed with the Children’s Sleep Habits Questionnaire (CSHQ).

Note: N = number of participants, Q = questionnaire.

In the studies, a wide variety of methods, such as self-reported questionnaires, interviews, or physician examination, were applied to verify the sleep problems. The majority of the studies utilized self-administered questionnaires, but five studies used standardized questionnaires (Kandel Depressive Symptoms Scale^13^, World Health Organization’s Health-related Quality of Life [WHOQOL-BREF]^13^, Chinese version of the Pittsburgh Sleep Quality Index^14^, and Children’s Sleep Habits Questionnaire^15, 27^. The definition of sleep problems and the methods used to verify juvenile injuries also widely varied among the papers. All the studies adopted self-reports, but 10 studies additionally utilized registered data^12–16, 23–27^.

For the methodological quality, we can see that five studies got 8 stars, because they are consistent with the result of the NOS evaluation standard. Besides, two studies got 7 stars because of no detailed description of the study design or statistical analysis. In addition, three studies only got 4 stars and the main deficiency was associated with different adjustments of confouding factors among the included studies.

Totally, the methodological quality of seven studies^12–16, 26, 27^ were considered of high quality, and three studies^23–25^ were regarded to be of poor quality on the basis of the NOS. The main deficiency is the selection bias related to insufficient adjustment of core factors and measurements based on self-reports (Supplemental Table S1).

### Overall meta-analysis results

**Figure 2:**
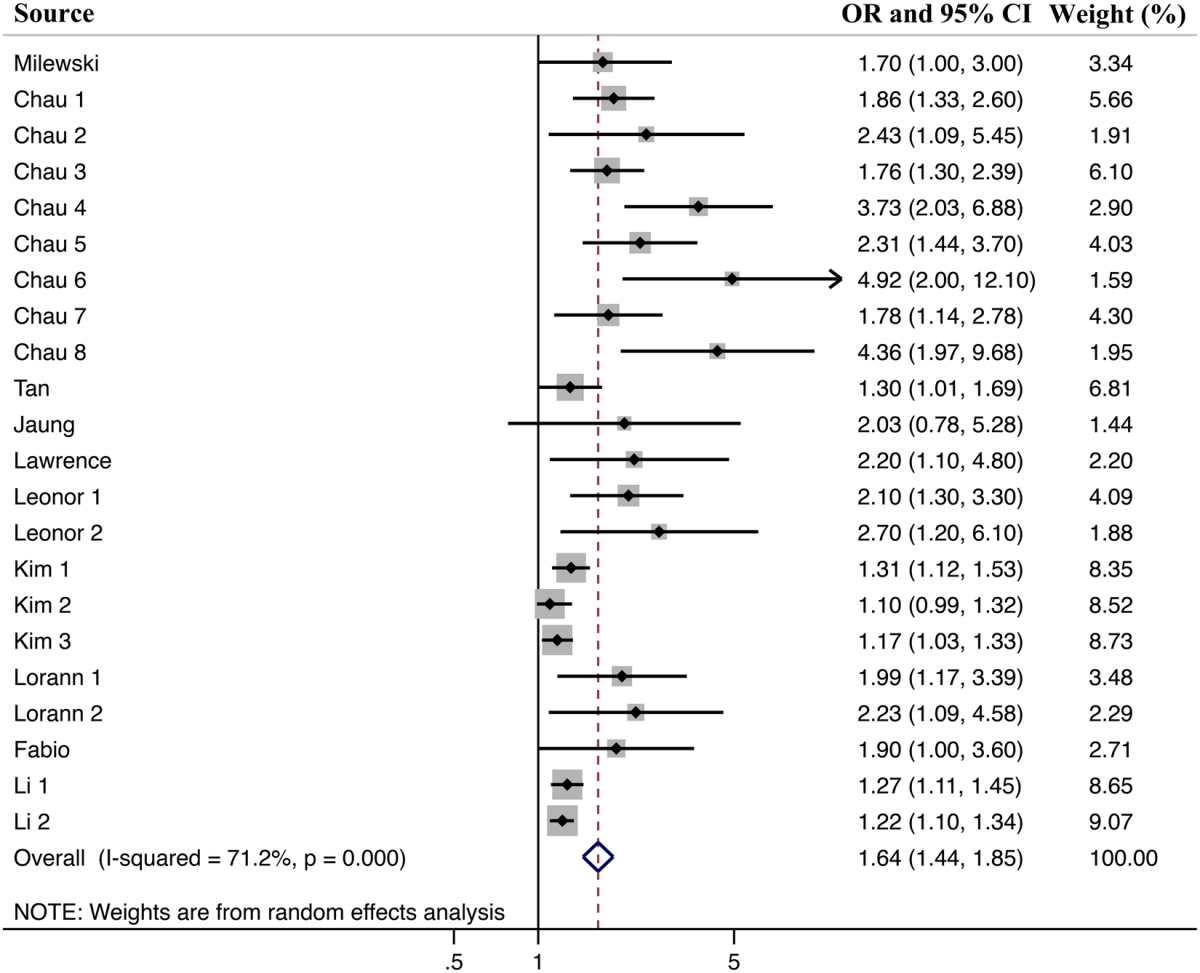
Overall meta-analysis results. Note: Meta-analysis of 10 studies showed that juveniles with sleep problems hold a higher risk of being injured than juveniles without sleep problems (OR: 1.64, 95% CI: 1.44–1.85), and with a moderate heterogeneity (I^2^ = 71.2%, P < 0.0001).

Meta-analysis of 10 studies^12–16, 23–27^ showed that juveniles with sleep problems hold a higher risk of being injured than juveniles without sleep problems (Fig. 2). The pooled OR was 1.64 (1.44 to 1.85), and the heterogeneity was significant (I^2^ = 71.2%, P < 0.0001). Thus, a random-effects model was used for pooled analysis.

### Subgroup analyses results

**Figure 3:**
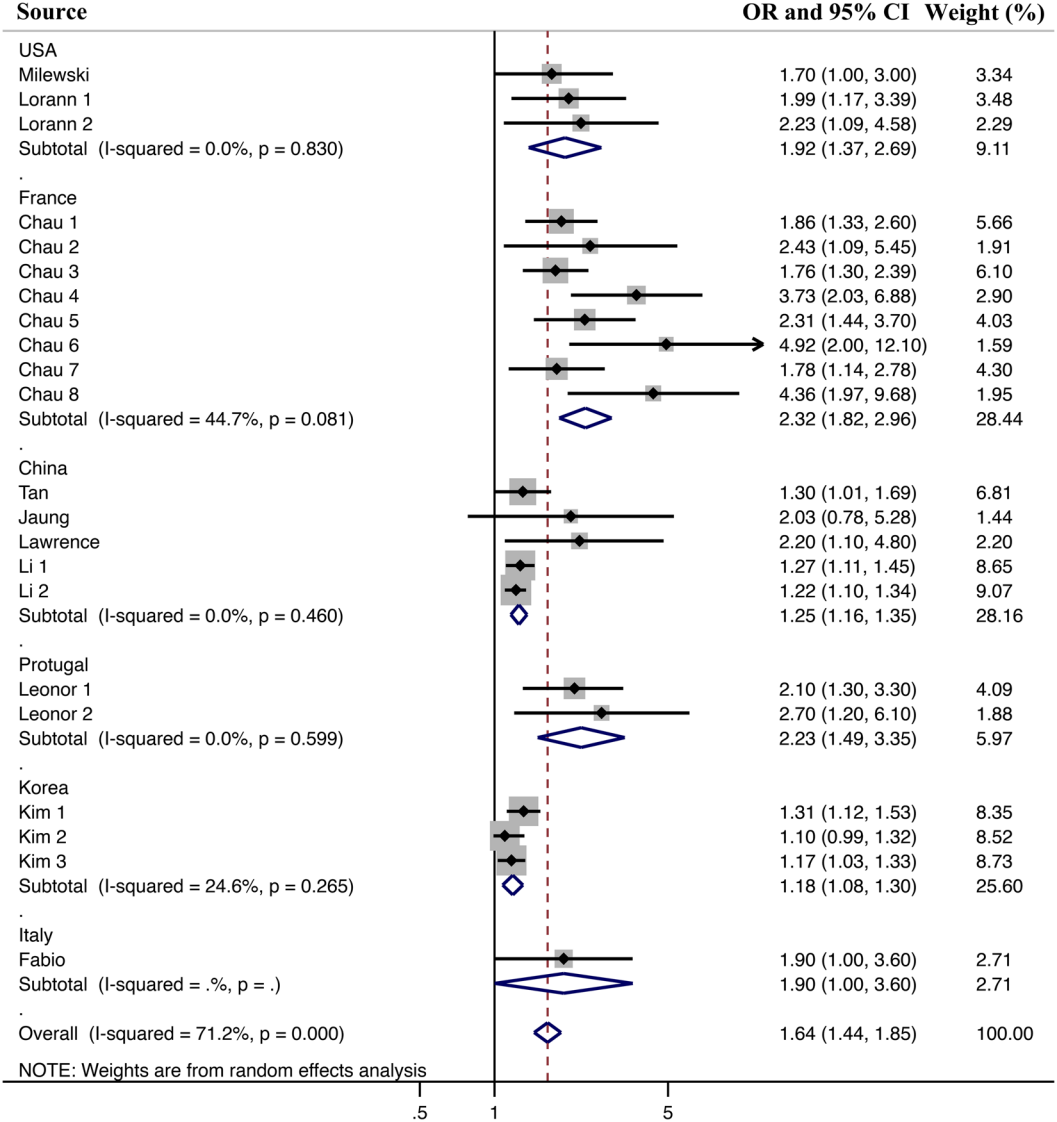
Subgroup meta-analysis for the effect of different countries. Note: All of the included studies were from USA, France, China, Protugal, Korea and Italy. Meta-analysis showed that juveniles from different countries with sleep problems still hold a higher risk of being injured than juveniles without sleep problems. The heterogeneity would be lower or zero, when juveniles were grouped by their own countries.

**Figure 4:**
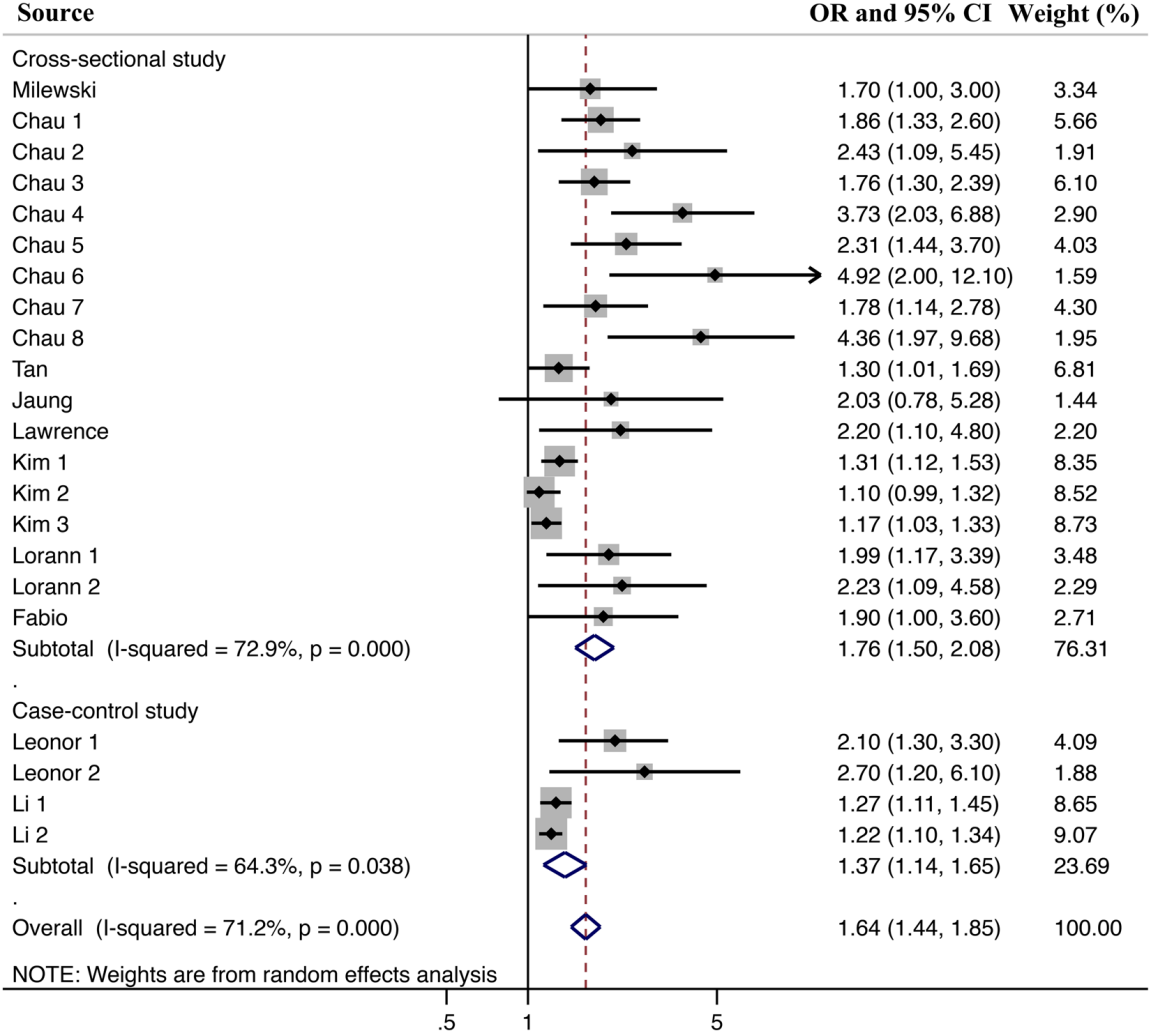
Subgroup meta-analysis for the effect of different study designs. Note: The included studies could be classified into Cross-sectional study and Case-control study. In the two subgroups divided by different study design, the Meta-analysis result still supported the points that juveniles with sleep problems hold a higher risk of being injured than juveniles without sleep problems. Besides, the heterogeneity of Meta-analysis only including case-control study would be lower than that of the Meta-analysis including all studies.

**Figure 5:**
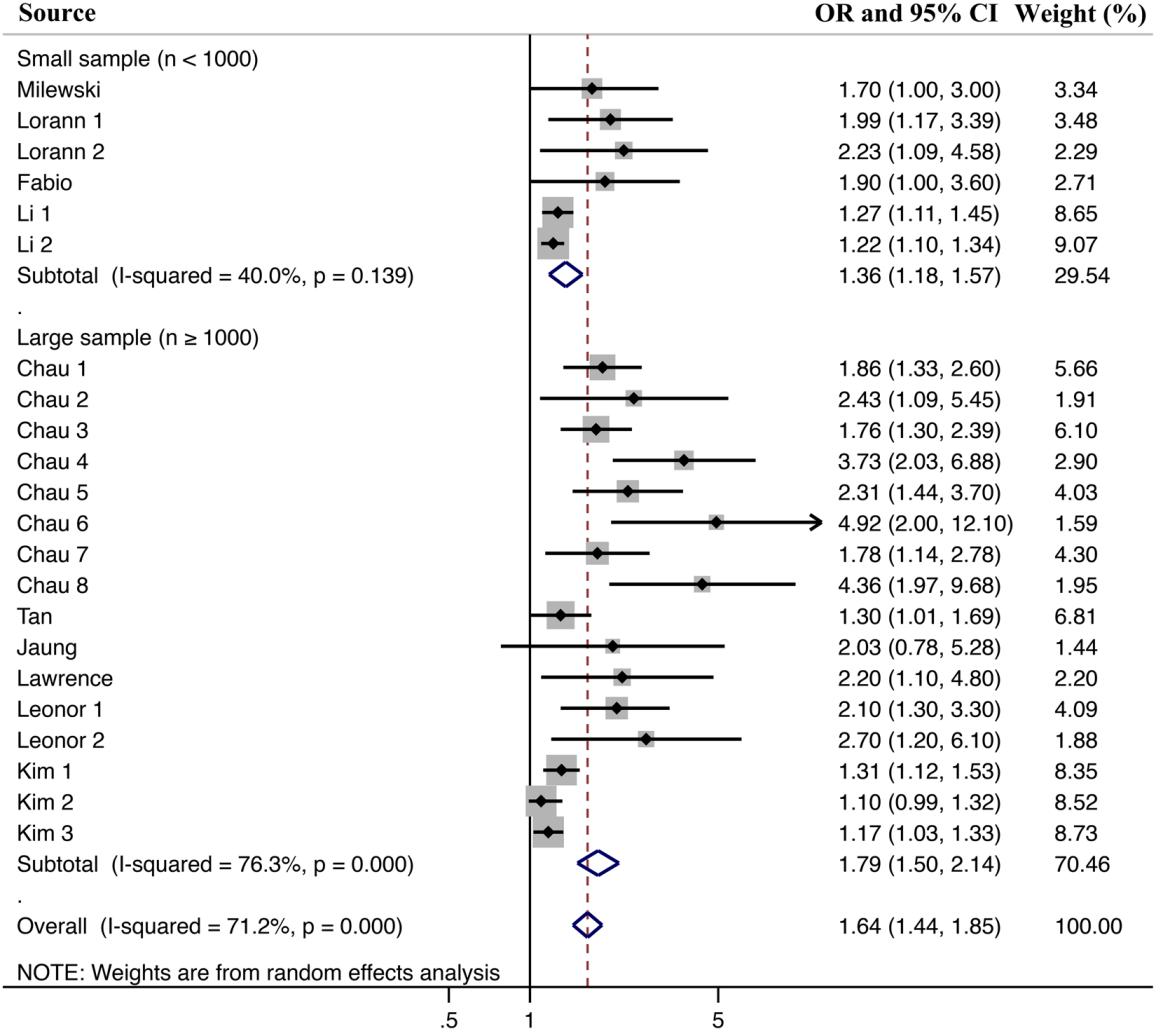
Subgroup meta-analysis for the effect of different sample size. Note: The sample size of the included studies is different. In our study, a sample size of lower than 1000 juveniles was considered as small sample size. Accordingly, a sample size of higher than 1000 juveniles was thought as large sample. In spite of different sample size, the meta-analysis still showed that juveniles with sleep problems still hold a higher risk of being injured than juveniles without sleep problems. For the study with small sample size, the heterogeneity would be lower.

In the subgroup analyses (Figs 3 and 4) of the different countries and study designs, we found that despite the different magnitudes, the findings on the association between sleep problems and injuries were consistent. Accordingly, the study design did not significantly contribute to the heterogeneity (all P values > 0.05). However, the heterogeneity can be explained by the subgroup analysis on the country of origin. For different volume of sample size, the Meta analysis result is still consistent with the overall result (Fig. 5). However, the polled results of studies with low sample size had a lower heterogeneity.

### Evaluation of publication bias

**Figure 6:**
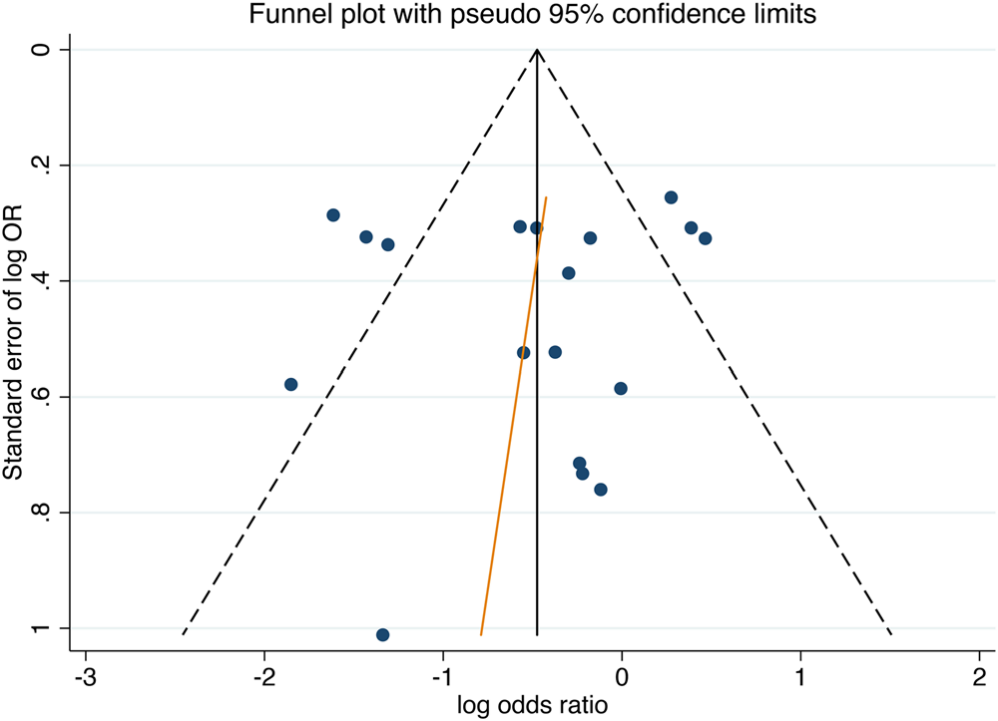
Funnel plot. Note: In this figure, a spot represents an included study. With the included studies, a regression line was drawn. It could be found that the spots surrounded the regression line did not present with a good symmetry. Therefore, the existence of potential publication bias was detected in our studys.

Publication bias was evaluated by the Egger’s test and then the funnel plot was graphed (Fig. 6). The Egger’s test result (*P = *0.02) indicated that there is certain publication bias in our study. For the funnel plot, each dots represents a study. It was showed that all of these dots distributed in an unsymmetrical way when referring to the graphed line. Therefore, the result of funnel plot also supported the idea that a publication bias was detected in our study. In our opinions, the most common reasons of the publication bias ought to be ascribed to the negative results not published in the previous literature.

## Discussion

The meta-analysis systematically and comprehensively evaluated the association between sleep problems and injury risk in 10 observational studies of juveniles worldwide. According to the main result, sleep problems cause 1.64 times higher risk of injury in juveniles than in those without sleep problems. In more detail, common sleep problems, such as insufficient sleep quantity or abnormal daytime sleepiness, could significantly increase the injury risk.

In recent years, an increasing number of people pay close attention to sleep problems and their influence, and preventive measures for sleep problems require further evaluation^28–30^. Future intervention studies with large samples may further consolidate understanding on how sleep-related injuries among juveniles might be prevented. Moreover, clinicians and other health-care providers working with parents should be aware of the risks and effects of sleep problems among juveniles^31–33^.

It was thought to be very valuable to explore whether these findings are applicable to the frequency or severity of injuries and the severity of sleep problems among juveniles. We also understood the mechanisms closely involved in how sleep problems affect injuries in juveniles^34^. Nevertheless, the association between sleep problems and injury frequency and severity in juveniles remains unclear because of the lack of studies examining such relationship. Hence, our understanding of gender differences, age, or types of sleep problems is insufficient, although some of these factors have been rarely investigated in other studies^35, 36^. Moreover, our subgroup analysis found that the high heterogeneity across studies was not potentially ascribed to the study design but partially to the country of origin. As we found, sleep difficulty resulted in lower risk of injury in China and Korea vs W Europe. It might be related to issues with self-report. Besides, it might also benefit from other reasons, such as different administration of the transportation. Thus, further research is needed to verify the findings of this meta-analysis with regard to the possible influence of age and sleep-problem severity in juveniles on the extensive consequences for preventive measures.

The study also holds some limitations that must be acknowledged before these findings are accepted. First, the studies varied in the degree of control for confounding factors, such as gender, age, ethnic background, socioeconomic status, and other health-related behavior. As such, the various studies were difficult to reconcile. Second, heterogeneity is another critical issue that must be given close attention because of this factor’s possible association with the population’s age, sleep-problem severity, and injury frequency. Third, the effect of publication bias should be considered. In this meta-analysis, all the included studies were conducted in juveniles from USA, China, France, Portugal, Korea, and Italy. Almost all the studies claimed that sleep problems held a similar relationship with injuries, and negative conclusions were not reported. Moreover, Egger’s regression test and visual analysis of the distribution of the relative risks by using a funnel plot suggested the existence of potential publication bias. Lastly, no cohort study was included in our study even after the systematic search of several databases. Therefore, additional studies with superior study designs should be conducted in the future.

## Conclusions

The meta-analysis showed that sleep problems were associated with an increased injury risk among juveniles. However, the confidence in the effect estimates may be low because of the limited number of participants, the various methodologies used, and the large number of possible confounding factors. Future prospective observational studies with larger sample sizes and longer follow-ups than those in the included works are needed. Additional studies that considered other sleep disorders and possible variations by gender, age, and race are still necessary.

## Electronic supplementary material

Supplemental Table S1
