# Supplementary material for: Sleep problems and injury risk among juveniles: A systematic review and meta-analysis of observational studies
Source: Sci Rep. 2017 Aug 29;7:9813. doi: 10.1038/s41598-017-10230-3 (PMC5575330; doi:10.1038/s41598-017-10230-3)
Supplement: Supplementary file 1 — Supplemental Table S1 [file 41598_2017_10230_MOESM1_ESM.doc]

**Sleep problems and injury risk among juveniles: A systematic review and meta-analysis of observational studies**

Yun-Bing Wang^1,2,*^, Zhen-Lang Guo^3,*^, Fan Zhang^4^, Yong Zhang^4^, Shu-Sheng Wang^5^, Yong Zhao^4^

^1^Graduate School, Chongqing Medical University, Chongqing, 400016, China;

^2^Department of Hepatobiliary Surgery, The Second Affiliated Hospital of Chongqing Medical University, Chongqing, 400010, China;

^3^The Second Clinical College, Guangzhou University of Chinese Medicine, Guangzhou, 510405, China;

^4^School of Public Health and Management, Chongqing Medical University, Chongqing, 400016, China;

^5^Department of Urology, Guangdong Provincial Hospital of Chinese Medicine, Guangzhou, 510120, China.

*These authors contributed equally to this work.

Correspondence and requests for materials should be addressed to Yong Zhao (Email: zhaoyong@cqmu.edu.cn) and Shu-Sheng Wang (Email: shusheng-wang@163.com).

**Supplemental Table S1. Methodological quality of the included studies (n = 10).**

| study | Item | | | | | | | | | | |
| --- | --- | --- | --- | --- | --- | --- | --- | --- | --- | --- | --- |
|  | 1 | 2 | 3 | 4 | 5 | 6 | 7 | 8 | 9 | 10 | Overall  score |
| Milewski et al. 2014^12^ | Y | Y | Y | Y | N | Y | Y | Y | N | Y | 8 |
| Chau et al. 2015^13^ | Y | Y | U | Y | Y | Y | N | Y | Y | Y | 8 |
| Tan et al. 2015^14^ | Y | Y | N | Y | U | Y | Y | Y | Y | Y | 8 |
| Jaung et al. 2009^15^ | Y | Y | Y | U | N | Y | Y | Y | Y | Y | 8 |
| Lam et al. 2007^23^ | Y | U | U | N | N | Y | U | Y | N | Y | 4 |
| Boto et al. 2012^24^ | Y | U | Y | U | Y | N | U | U | N | Y | 4 |
| Kim et al. 2015^16^ | Y | Y | Y | Y | U | U | Y | N | Y | Y | 7 |
| Stallones et al. 2006^25^ | Y | Y | N | N | Y | U | U | N | N | Y | 4 |
| Pizza et al. 2010^26^ | Y | Y | Y | N | N | U | Y | Y | Y | Y | 7 |
| Li et al. 2008^27^ | Y | Y | U | Y | Y | Y | U | Y | Y | Y | 8 |

Note: 1. Target population with representative sample;

2. Study participants were recruited appropriately?

3. Adequate sample size;

4. Detailed study subjects and setting;

5. Data analysis were performed with sufficient coverage;

6. The measurement of the condition were evaluated with standard criteria;

7. Condition was measured reliably;

8. Appropriate statistical analysis was conducted;

9. Some important confounding factors were identified;

10. Subpopulations were identified using objective criteria;

Y, yes; U, unclear; N, no.
